# Anthrax

**Published:** 1877-11-20

**Authors:** Jacob Van Valkenburgh

**Affiliations:** Sharon, N. Y.


### ANTHRAX.

BY JACOB VAN VALKENBURGH, M. D., SHARON, N. Y.

Anthrax is a tumor of a red, shining appearance, commonly known as carbuncle. The larger tumors of this class are full of holes, and may be defined to be a local destruction of the cutaneous and areolar tissue depending upon an accumulation of morbific material in the part, of a malignant character, usually larger than a boil and less conical. They vary in size from a hickory nut to a dinner plate, presenting a flattened surface of a livid red or purplish appearance. The pain is circumscribed and of a burning character. The usual symptoms of fever of a typhoid grade are shown by the constitutional disturbance. As the tumor increases in size, one or several vesicles form on the surface, from which oozes a thin, acrid, fœtid, dark-colored fluid. Its location is most frequently over the bony surfaces—as the scapula, spinal column, cranium, dorsum of the feet and hands. It implies a vitiated and debilitated condition of the general system. If this were not the case it would have been a boil. The obstruction in the cellular and cutaneous tissue that causes a boil attended with a mild inflammatory reaction, or symptomatic fever of the inflammatory grade, would be an anthrax in a worn out, vitiated system, attended with a feeble reaction, commonty known as the typhoid grade.

The local obstruction that occurs porduces irritation; the *vis medicatrix* sets up a vital action known as inflamation, for restoring the parts affected; this in nearly the whole of the tumor, fails, in consequence of the debilitated state of the general system, or from the character of the obstruction, and, consequently, congestion takes place, followed by suppuration, gangrene, and death in many cases.

About the only mistake liable to be made in diagnosticating is a boil. In this there is a general appearance of health, a conical tumor, which, after suppuration, has a "central core;" whereas, in carbuncle, the tumor is larger and flatter; vesicles appear on the tumor, and it has no "core." The size, color, etc., will serve sufficiently to distinguish it from the boil. Carbuncle appears in old worn-out constitutions, caused by damp place of living, or any cause resulting in great nervous prostration; all or any produce that enfeebled condition, by which the vital powers cannot remove accumulated *materies morbi* from the system. The prognosis depends on the general stamina of the patient, the malignancy of the tumor, the skill of the practitioner, and the remedies used.

TREATMENT.

Apply to the carbuncle a hot slippery-elm poultice, wet with the following solution:

R. Glyceriti Acidi Carbolici dr iv.
Aquaæ Puræ. oz v. Misce

Change the poultice often. Do not let it become cold. If slippery-elm connot be obtained, use the next best, the flaxseed poultice, and the solution above given. Give internally the following cathartic, repeating every four hours until it operates:

R. Pulv. Jalapæ et Sennae Comp.

" Leptandriæ, aa dr. j.

Sodæ Bicarb. grs xx.

Aquæ Bullientis, oz iv.

Let stand till cold, and take one half every hour.

After the operation of the cathartic, give:

R Potassæ Acetatis. dr ij scru. ij.

Aquæ Puræ, oz iv.

Misce: Teaspoonful every three hours.

As soon as pus is formed in the tumor, apply the knife early and freely, making crucical incissions through its length and breadth, that the putrid mass may have every opportunity to escape, as it is liable to be absorbed, pyæmia taking place, and death ensuing. As soon as the contents of the carbuncle are evacuated, apply to the cavity:

R. Glyceriti Acidi Carbolici, dr v to dr x

Aquæ, oz. iv.

Misce.

Use this solution as strong as the patient can bear, every time the poultice is changed. The carbolic acid is a stimulant, disinfectant, and *anti-septic* and is one of the best remedies for external congestion and gangrene that we posess.

As our object is to get rid of the tumor as speedily as possible, we must continue to poultice, and also to remove all sloughs with scissors or knife, which can be easily done by taking hold of the detached end with small forceps. I sometimes add some grated carrot root to the poultice, with the happiest results. As soon as the congested portion of the anthrax is sloughed out, and the healing process commenced, use the following carbolic salve: Take four ounces of basilicon oinment, melt with gentle heat, and add twenty grains of carbolic acid previously dissolved in an ounce and half of glycerine, stirring till cold. Spread on linen, and retain with a bandage if necessary. Some practitioners use caustic potash and charcoal poultice, with good results, in carbuncle. The cutaneous surface should be sponged off and dried with a flannel cloth. A good nutritious diet should be used to keep up the strength of the patient—such as beef tea, oyster soup, etc. The medical treatment must be supporting, and for this would use:

R. Tr. Ferri Chloride, gtts. xx.
Aqua, oz j
Mix. Take dr j. three times a day; or,
R Quiniae Sulph., dr ss.
Divide in 15 powders, and give one three times a day; or,
R Tr. Cinchonæ Comp., oz ij.
Two teaspoonfuls in water, three times a day.

The patient must have rest at night, and for this must use some opiate or nervine, as the idiosyncrasy requires; as opium, morphine, chloral hydrate, chloroform, bromide of potassum, valerian, or assafœtida.

If the patient becomes much exhausted we must resort to stimulants—such as Xanthoxylum, ale, porter, etc.—*Med. Eclectic.*

